# Over-expression of Toll-like receptor 2 up-regulates heme oxygenase-1 expression and decreases oxidative injury in dairy goats

**DOI:** 10.1186/s40104-016-0136-2

**Published:** 2017-01-09

**Authors:** Shoulong Deng, Kun Yu, Wuqi Jiang, Yan Li, Shuotian Wang, Zhuo Deng, Yuchang Yao, Baolu Zhang, Guoshi Liu, Yixun Liu, Zhengxing Lian

**Affiliations:** 1 State Key Laboratory of Stem Cell and Reproductive Biology, Institute of Zoology, Chinese Academy of Sciences, Beijing, 100101 China; 2 Laboratory of Animal Genetics and Breeding, College of Animal Science and Technology, China Agricultural University, Beijing, 100193 People’s Republic of China; 3 National key Lab of Agrobiotechnology, College of Biological Sciences, China Agricultural University, Beijing, 100193 People’s Republic of China; 4 Department of Animal Science, Oklahoma State University, Stillwater, OK 74078 USA; 5 College of Animal Science and Technology, Northeast Agricultural University, Harbin, 150030 People’s Republic of China; 6 State Oceanic Administration, Beijing, 100860 People’s Republic of China

**Keywords:** Haem oxygenase, Nrf2 signal pathway, Toll-like receptor 2, Transgenic goats

## Abstract

**Background:**

Mastitis, an infection caused by Gram-positive bacteria, produces udder inflammation and oxidative injury in milk-producing mammals. Toll-like receptor 2 (TLR2) is important for host recognition of invading Gram-positive microbes. Over-expression of *TLR2* in transgenic dairy goats is a useful model for studying various aspects of infection with Gram-positive bacteria, in vivo.

**Methods:**

We over-expressed *TLR2* in transgenic dairy goats. Pam3CSK4, a component of Gram-positive bacteria, triggered the TLR2 signal pathway by stimulating the monocytes-macrophages from the *TLR2*-positive transgenic goats, and induced over-expression of activator protein-1 (AP-1)*,* phosphatidylinositol 3-kinase (PI3K) and transcription factor nuclear factor kappa B (NF-κB) and inflammation factors downstream of the signal pathway.

**Results:**

Compared with wild-type controls, measurements of various oxidative stress-related molecules showed that *TLR2*, when over-expressed in transgenic goat monocytes-macrophages, resulted in weak lipid damage, high level expression of anti-oxidative stress proteins, and significantly increased mRNA levels of transcription factor NF-E2-related factor-2 (Nrf2) and the downstream gene, heme oxygenase-1 (HO-1). When Pam3CSK4 was used to stimulate ear tissue in vivo the HO-1 protein of the transgenic goats had a relatively high expression level.

**Conclusions:**

The results indicate that the oxidative injury in goats over-expressing *TLR2* was reduced following Pam3CSK4 stimulation. The underlying mechanism for this reduction was increased expression of the anti-oxidation gene *HO-1* by activation of the Nrf2 signal pathway.

## Background

Mastitis, an inflammatory and oxidative stress disease of udders, is caused by pathogenic bacteria. The disease is common in goats and other dairy animals. Mastitis reduces the quantity and quality of milk. Gram-positive bacteria such as staphylococcus, streptococcus and corynebacterium cause mastitis. *Staphylococcus aureus* is the main zoonotic agent of mastitis [1]. During infection, the innate immune system of the host animal provides the first line of defense in pathogen recognition, and pattern recognition receptors (PRRs) are necessary for this process to occur. Toll-like receptor (TLR) 2 plays a critical role in recognition of the conserved portion of Gram-positive bacteria [2], and it is known that mastitis can increase expression of the *TLR2* gene in mammary glands [3]. Genetic diversity in the *TLR2* gene is a risk factor for staphylococcus infection [4], and *TLR2−/−* mice are susceptible to *S. aureus* [5]. The *S. aureus* cell wall components (e.g., peptidoglycan, lipoteichoic acid and lipoproteins) are recognizable by TLR2 [6]. Pam3CSK4, a synthetic bacterial lipoprotein, is an agonist for TLR2 [7].

TLR2 is widely expressed in all types of goat immune cells and epithelial cells, and is highly expressed in peripheral blood monocytes. TLR2 activation relies mainly on the MyD88 signaling pathway, which activates the NF-κB pathway and mitogen-activated protein kinase (MAPK) pathway. Phosphorylated MAPK activates the transcription factor activator protein-1 (AP-1) and activation of the PI3K/Akt signaling pathway. Expression of inflammatory cytokines such as tumor necrosis factor α (TNF-α), interleukin (IL)-1β, IL-6, IL-8, chemokines and nitrous oxide (NO) can all induce immune responses.

In mastitis, oxidative stress results from infection with pathogenic bacteria. Such infections in udders trigger host immune responses that induce production of large amounts of reactive oxygen species (ROS) in host cells [8]. Transcription factor NF-E2-related factor-2 (Nrf2) plays a critical role in anti-inflammation and oxidation stress, induces anti-oxidation proteins and expression of the type II detoxification enzyme, and triggers expression of the downstream target genes catalase (CAT), superoxide dismutase (SOD), glutathione S-transferase alpha 1 (GSTα1), quinone oxidoreductase 1 (NQO1), γ-glutamylcysteine synthetase (γ-GCS) and heme oxygenase-1 (HO-1). Some cellular signaling pathways such as PI3K and MAPK regulate activation of the Nrf2 antioxidative response element pathway [9]. Oxidative stress activates NF-κB, triggers inflammatory responses, up-regulates expression of target genes such as cyclooxygenase-2 (COX-2), inducible nitric oxide synthase (iNOS) and the oxidation enzyme NADPH, and facilitates prostaglandin, ROS and NO production, while intensifying the oxidative stress response. Crosstalk between Nrf2 and NF-κB pathways cooperatively regulates the oxidative stress response [10]. HO-1 and its catalytic products enhance the innate protection system of the host [11]. The *HO-1* gene contains AP-1, NF-κB, Nrf2, which are regulatory structural sites. IL-10 and TLR pathways are involved in HO-1 regulation [12].

The expression level of TLR2 is closely associated with lipid oxidation and inflammation reactions [13], and also plays an important role in triggering innate and adaptive immune responses against infection, thereby protecting the host from disease. In this study, we over-expressed the *TLR2* receptor in laso-shan dairy goats. Through stimulation with the Pam3CSK4 ligand from Gram-positive bacteria, we observed the effect of TLR2 on the immune response. We discuss the mechanism whereby TLR2 regulates anti-oxidative stress.

## Methods

### Production of transgenic goats over-expressing toll-like receptor 2

**Fig. 1:**
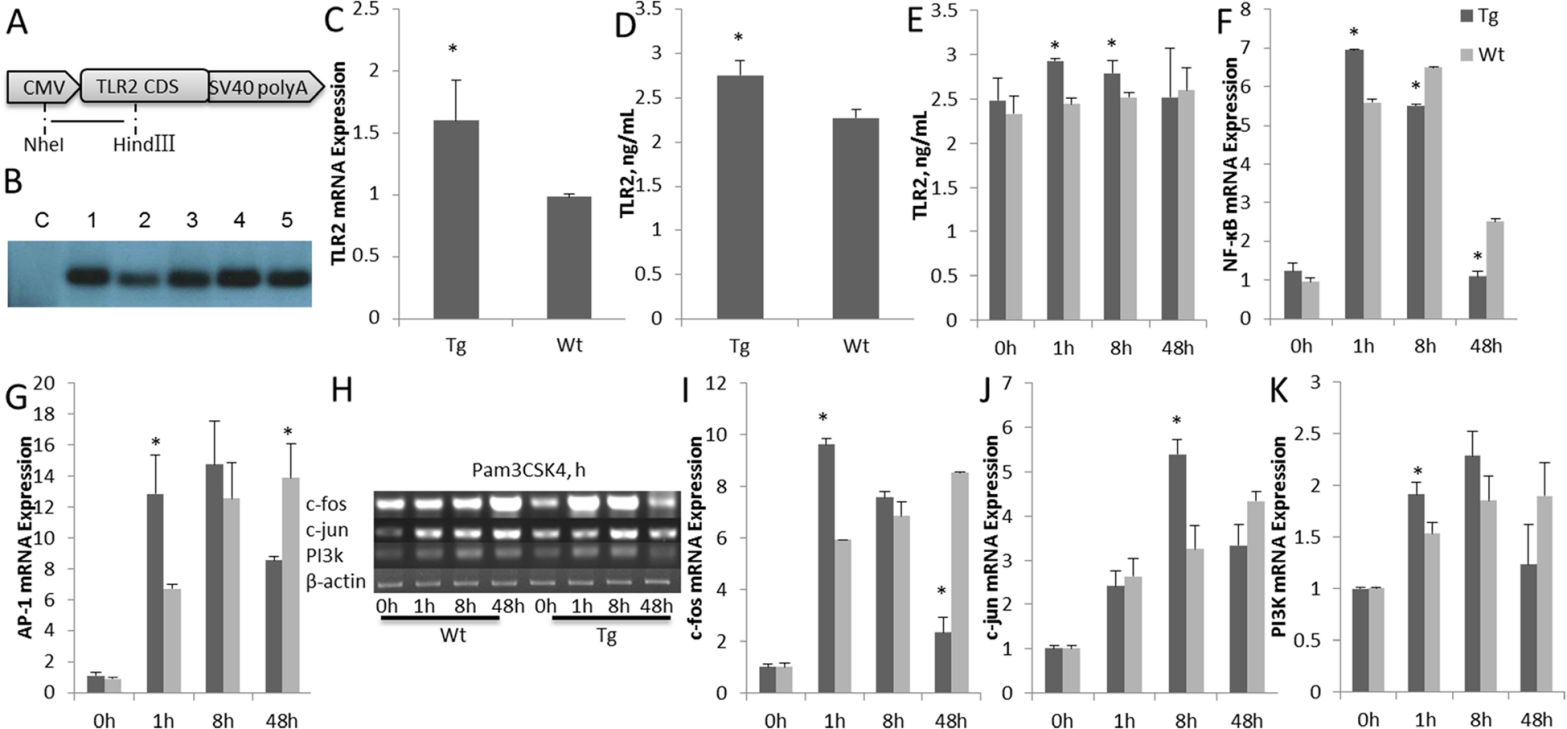
Overexpression of TLR2 in goats. **a** Structure of TLR2 plasmids. **b** TLR2-positive goats were detected by Southern blot analysis. Lanes: **c**, control (Wt); 1–5, genomic DNA. **c** *TLR2* expression in monocytes-macrophages was detected by qRT-PCR. **d** ELISA analysis of TLR2 expression in goat sera. **e** ELISA analysis of TLR2 expression in monocytes-macrophages. **f** Expression of *NF-κB* and **g** *AP-1* was detected by qRT-PCR. (**h**), (**i**), (**j**) and (**k**). The gene expression levels of *c-Fos*, *c-Jun* and *PI3K* were quantified by RT-PCR. Tg: Transgenic goat, Wt: wild type. The results are expressed as the means ± SEM. ^*^ *P* < 0.05 in Tg versus Wt groups

Transgenic goats were produced by microinjecting plasmid constructs into fertilized goat eggs (Fig. 1a). The transformed exogenous *TLR2* genes in the offspring were analyzed by Southern blotting. Genomic DNA was extracted from ear tissue, and a gene-specific digoxigenin-labeled probe (Roche Diagnostics, Mannheim, Germany) was generated by PCR amplification with the following primer pair, oTLR2, forward: 5′-TTC TCC CAC TTC CGT CTC-3′; reverse: 5′-CCC TAT CTC GGT CTA TTC TT-3′, resulting in a 618-bp fragment. Genomic DNA was isolated from ear tissue and digested with *Nhe*I and *Hin*dIII (NEB, Beverly, MA, USA) [14].

### Isolation purification and culturing of goat monocytes-macrophages

Each group had 5 transgenic goats. Peripheral blood mononuclear cells (PBMCs) were isolated from the peripheral blood of 6-month-old transgenic goats using goat lymphocyte separation medium (TBD, Tianjin, China). The PBMCs containing thrombocytes, lymphocytes, and monocytes were washed twice with Hanks’ buffer (Invitrogen, Beijing, China). The harvested cells at a concentration of 2 × 10^7^ cells/well were seeded in 6-well plate. The plates were incubated at 37 °C in an incubator with a 5% CO_2_ for 2 h and then the non-adherent cells (most of lymphocytes) were washed out with PBS. The adherent cells were cultured with RPMI1640 (Gibco, Grand Island, NY, USA) medium containing 10% FBS (Gibco). After 24 h of incubation and 3 times washing, the cells were digested with 0.02% EDTA for 5 min, and then the cells were harvested. The cells (1 × 10^5^) were seeded in each well of a 6-well plate with three repeats for each group. RPMI1640 medium containing 10% FBS was changed every 24 h. After 48 h of incubating and washing away non-adherent cells, the adherent cells were mainly monocytes-macrophages [15, 16]. Monocytes-macrophages were stimulated by Pam3CSK4 (1 μg/mL) (InvivoGen, San Diego, CA, USA) [17]. Cell suspensions were collected at 0, 1, 8 and 48 h post-stimulation. RNA was extracted from adherent cells using TRIzol plus RNA purification kit (Invitrogen, Carlsbad, CA, USA) and treated with RNase-free DNase (Promega, Madison, WI, U.S.) per the manufacturer’s instructions. cDNA was synthesized with a Reverse Transcription kit (Promega, Madison, WI, USA).

### Reverse transcription (RT) and quantitative real-time (qRT)-PCR

cDNA from Pam3CSK4-stimulated monocytes-macrophages was obtained at different time points. The abundance of *TLR2*, *AP-1*, *NF-κB*, *Nrf2*, *SOD1*, *CAT*, *GSTα1*, *NQO1* and *HO-1* mRNA molecules was measured by qRT-PCR. The gene expression levels of *c-Fos*, *c-Jun* and *PI3K* were quantified by RT-PCR. *β-actin* was used as an internal standard. PCR products were analyzed by agarose gel electrophoresis. Primer sequences are shown in Table 1. qRT-PCR was performed using a Realtime Master Mix SYBR Green kit (Tiangen, China) using MX300P (Stratagene).

**Table 1:** The primer sequences

Primer	Forward (5'→3')	Reverse (5'→3')
TLR2	TGCTGTGCCCTCTTCCTGTT	GGGACGAAGTCTCGCTTATGAA
AP-1	GAAGGAAGAGCCGCAGACG	CCACCTGTTCCCTGAGCATA
NF-κB	TTCTCCAAATGGCTGAAGGTA	TTGTTTGAGGGCCATAAGGAT
Nrf2	CCAACTACTCCCAGGTAGCCC	AGCAGTGGCAACCTGAACG
c-Fos	GAGGACCTTATCTGTGCGTGAA	GGTTAATTCCAATAACGAACCCAAT
c-Jun	AAGATGGAAACGACCTTCTACGA	TTGATCCGCTCCTGGGACT
PI3K	CCGACAGTGAGCAACAAGC	AGGAGGCGGCATCACAATC
CAT	TTTTCTGCTGAAGCCCTATGA	GTCCTTTCAGGGAGAATGGTG
GSTα1	TGAGCCAAGTGGGAGACAGAC	ATCCAGGTCTTCTGGTTGTTCTAT
SOD1	TCTGCGTGCTGAAGGGTGA	CTTTGGCCCACCGTGTTTT
HO-1	ACACCCAGGCGGAGAATG	CTCCTGGAGTCGCTGAACATAG
NQO1	AACTTCAATCCCGTCATCTCC	CCTTTCAGGATGGCAGGGACT
β-actin	AGATGTGGATCAGCAAGCAG	CCAATCTCATCTCGTTTTCTG

### Enzyme-linked immunosorbent assays (ELISAs)

During the TLR2 receptor agonist experiments, cell culture supernatants from Pam3CSK4-stimulated monocytes-macrophages were collected at different time points (0, 1, 8, and 48 h). The concentrations of TLR2, TNF-α, IL-1β, IL-8, IL-12p40, IL-4 and monocyte chemoattractant protein-1 (MCP-1) in the supernatants were measured using ELISA kits (CUSABIO, Hubei, China). Sera were obtained to detect TLR2 concentration using ELISA kit. Blood samples were collected from goats and allowed to clot at 37 °C for 30 min. Sera were separated immediately by centrifugation at 3,000 r/min for 15 min. All experimental operations were performed according to the kit instructions.

### Oxidative stress measurements

Pam3CSK4-stimulated monocytes-macrophages suspensions were collected at different time points (0, 1, 8, and 48 h). The activities of iNOS, SOD, CAT, GSH, COX-2, NADPH oxidase and malondialdehyde (MDA) contents were examined by spectrophotometry in accordance with the manual supplied with the detection kit (Nanjing Jiancheng, China).

### Immunocytochemical staining

The ears of three 8-month-old transgene-positive goats were injected intradermally with 100 μL of 3 mg/mL Pam3CSK4, after which the tissues were collected at 1, 8, and 48 h [18, 19]. Samples were fixed with 4% paraformaldehyde and embedded in paraffin. Hematoxylin and eosin staining was used to investigate inflammatory responses and immunohistochemistry was used to detect HO-1 protein expression (Abcam, ab13248, Cambridge, UK). Six fields from each slide were randomly selected. Optical densities were quantified by scanning densitometry and expressed in arbitrary units determined by Image J software (NIH, USA).

### Statistical analyses

Individual experiments were repeated three times. Statistical tests to determine differences of *TLR2* mRNA expression between two groups were performed with one-way ANOVA followed by Tukey’s HSD using Statistical Analysis System (SAS Institute, Cary, NC, USA). A repeated measures ANOVA with SAS proc GLM followed by Tukey’s HSD post-hoc tests were performed to determine the statistical significance between the relative groups in different time points. All data were expressed as mean ± SEM. Differences were considered to be significant when *P* < 0.05.

## Results

### Toll-like receptor 2 over-expression triggers activation of down-stream transcription factors

The microinjection technique successfully generated *TLR2* over-expression in goats. Transfected goats positive for exogenous *TLR2* were identified by Southern blot analysis of genomic DNA (Fig. 1b), the positive rate was 9.82% (Table 2). Figure 1c shows that *TLR2* expression in the transgenic goats was higher than that of the wild-type (Wt) animals (*P* < 0.05). The expression of TLR2 protein in sera in the transgenic group was significantly higher than that in the Wt group (*P* < 0.05) (Fig. 1d).

**Table 2:** Microinjection production of over-expression of *TLR2* gene in goats

No. of donors	No. of microinjections	No. of embryo transfer recipients	No. of lambs	Southern positive rate, %
11	127	30	22	22.73%

ELISA was performed to measure the TLR2 expression levels in the Pam3CSK4-stimulated monocytes-macrophages. TLR2 expression levels in the transgenic group were significantly higher than those of the Wt group at 1 and 8 h post-stimulation (*P* < 0.05) (Fig. 1e). These results confirmed that TLR2 expression of the monocytes-macrophages of the transgenic group was up-regulated after Pam3CSK4 stimulation. Next, qRT-PCR was conducted to determine *NF-κB* and *AP-1* mRNA expression levels. In this experiment, *NF-κB* expression in the transgenic group was significantly higher than that in the Wt group at 1 h, and reduced at 8 h post-stimulation compared with Wt (Fig. 1f). These results show that TLR2 triggered activation of the NF-κB signal pathway, with *NF-κB* expression levels decreasing at an earlier stage than those of Wt. *AP-1* expression in the transgenic group was significantly higher than that of the Wt group at 1 h post- stimulation, reached a peak at 8 h, while its expression in Wt group continued to increase over time (Fig. 1g). The expression levels of *c-Fos*, *c-Jun* and *PI3K* were similar as *AP-1* (Fig. 1h, i, j and k).

### Toll-like receptor 2 over-expression improves expression of the anti-inflammatory factor

**Fig. 2:**
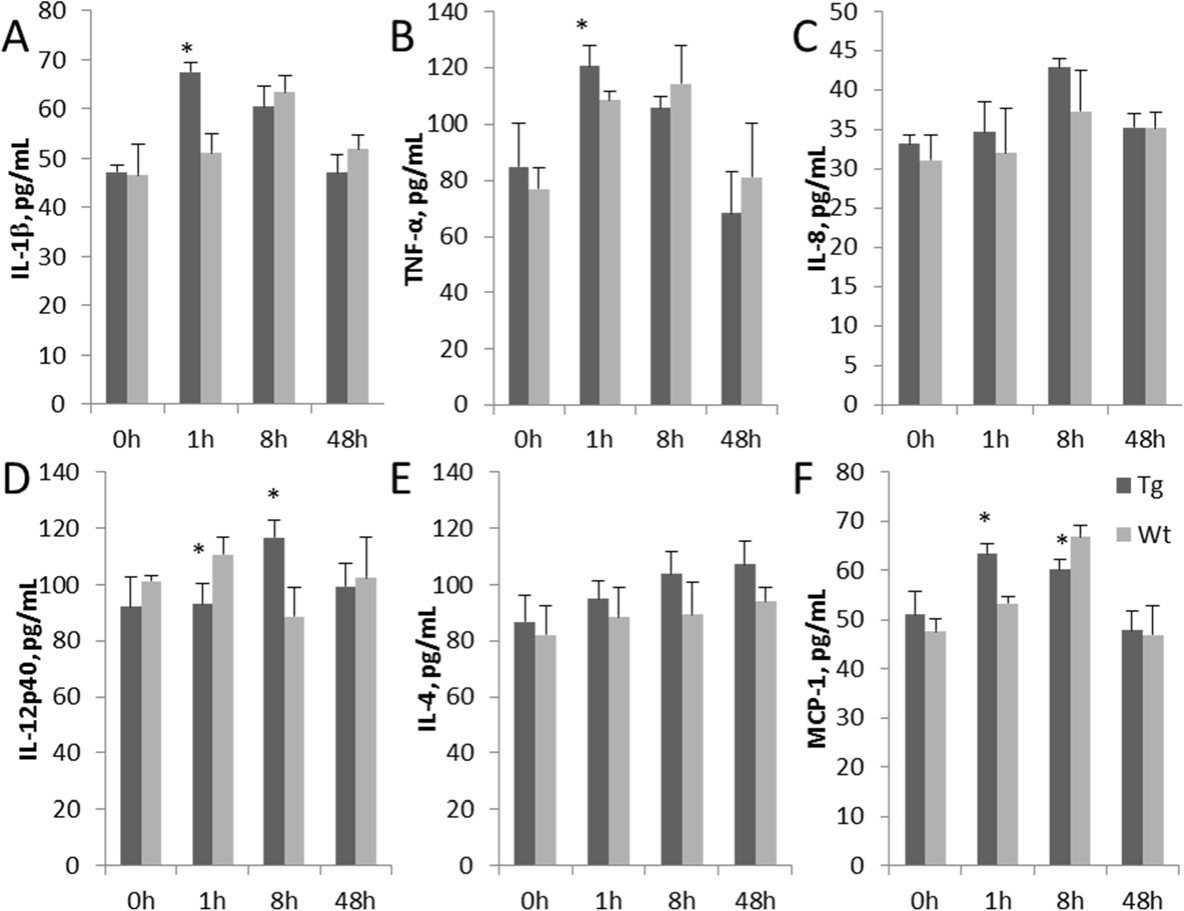
ELISA testing of inflammatory cytokines changes in TLR2 monocytes-macrophages under Pam3CSK4 stimulation. **a**, **b**, **c**, **d** and **e** show the effects of IL-1β, TNF-α, IL-8, IL-12p40 and IL-4 immune factors, respectively. **f** shows MCP-1 expression. Tg: Transgenic goat, Wt: wild type. The results are expressed as the means ± SEM. ^*^ *P* < 0.05 in Tg versus Wt groups

After Pam3CSK4 stimulation of goat monocytes-macrophages, expression of the pro-inflammatory factors IL-1β and TNF-α were similar (Fig. 2a and b). At 1 h post-stimulation, the expression level of the transgenic groups was significantly higher than that of the Wt group (*P* < 0.05). Furthermore, expression in the transgenic group both IL-1β and TNF-α peaked at 1 h, while in the Wt group the peak occurred at 8 h, IL-8 expression in the transgenic and Wt groups was not significantly different at the different time points (Fig. 2c). Expression of the type Th1 cytokine IL-12p40 in the Wt group peaked at 1 h and was significantly higher than that of the transgenic group; it subsequently decreased and then increased at 48 h post-stimulation. The expression level of the transgenic group peaked at 8 h, which was slower than that of the Wt cells (Fig. 2d). This result shows that over-expressed TLR2 retarded the expression of type Th1 cytokines. IL-4 expression continuously increased in transgenic group during the experiment (Fig. 2e). For MCP-1 expression (Fig. 2f), the transgenic group was significantly higher than the Wt group at 1 h (*P* < 0.05), but significantly lower at 8 h post-stimulation.

### Weakens oxidative lipid injury in monocytes-macrophages

**Fig. 3:**
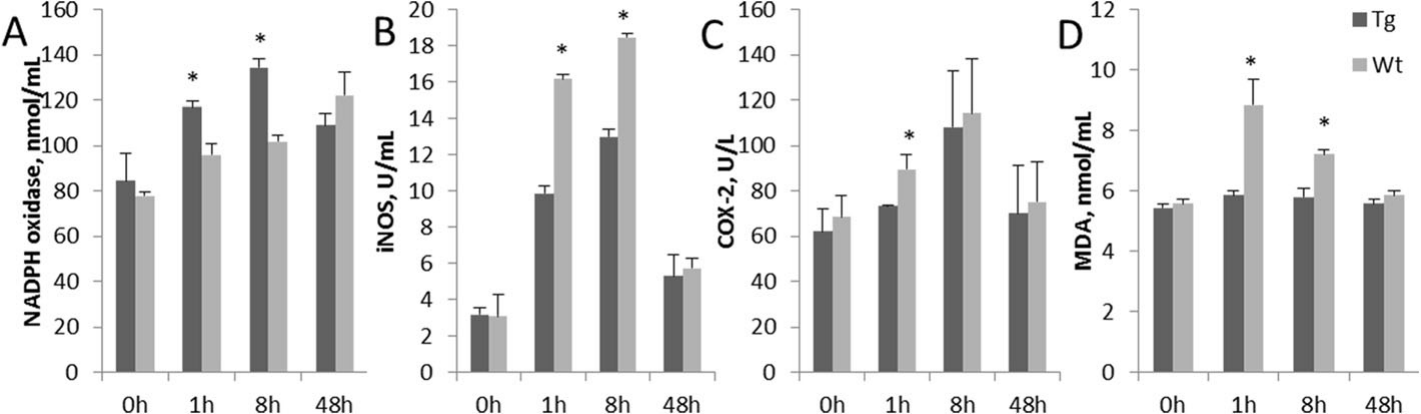
Over-expressed TLR2 confers weak lipid oxidative injury. The activities of NADPH oxidase, iNOS, COX-2 and MDA content (**a**, **b**, **c** and **d**, respectively). Tg: Transgenic goat, Wt: wild type. The results are expressed as the means ± SEM. ^*^ *P* < 0.05 in Tg versus Wt groups

In this experiment, after Pam3CSK4 stimulation of goat monocytes-macrophages, the NADPH oxidase expression level in the transgenic group was higher than that of the Wt group at 1 h and 8 h post-stimulation (*P* < 0.05) (Fig. 3a). iNOS expression in the transgenic and Wt groups showed similar trends (Fig. 3b), though the level in the transgenic group was significantly lower than that of the Wt group at 1 h and 8 h post-stimulation (*P* < 0.05). COX-2 is involved in the stress reaction against pathological conditions. In this experiment, the COX-2 expression level reached a peak at 8 h in both group (Fig. 3c). At 1 h, the level in the Wt group was significantly higher than that of the transgenic group (*P* < 0.05). The expression level of the over-oxidative lipid product (MDA) in the transgenic group was relatively stable over time unlike that of the Wt group (Fig. 3d), and was also significantly lower than that of the Wt group at 1 and 8 h post-stimulation (*P* < 0.05).

### Over-expressed toll-like receptor 2 retains anti-oxidative protein activity in goat monocytes-macrophages

**Fig. 4:**
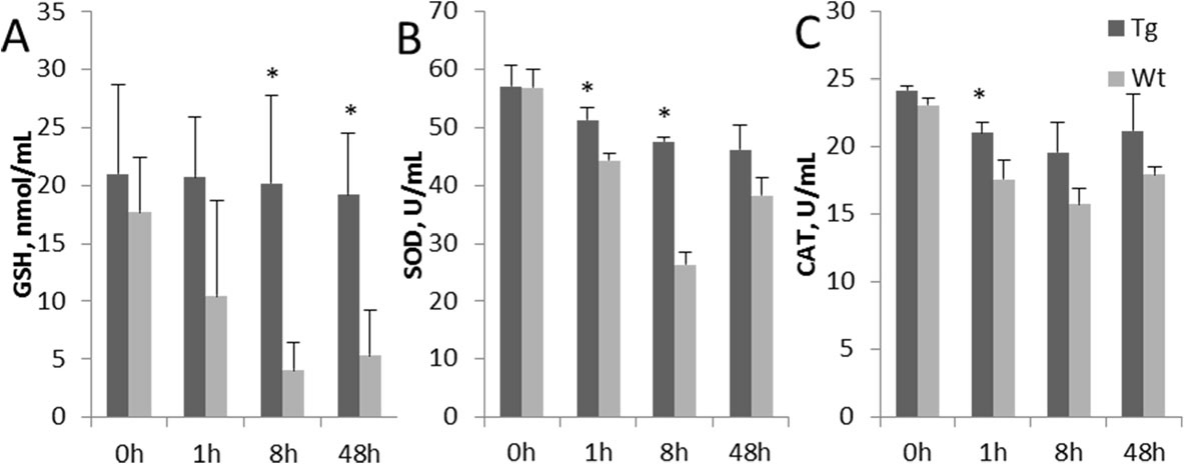
Oxidative stress protein expressions in goat monocytes-macrophages. The activities of GSH, SOD and CAT are shown in **a**, **b** and **c**, respectively. Tg: Transgenic goat, Wt: wild type. The results are expressed as the means ± SEM. ^*^ *P* < 0.05 in Tg versus Wt groups

In this experiment, after Pam3CSK4 stimulation of goat monocytes-macrophages, the GSH content was maintained at a stable level in monocytes-macrophages the transgenic group (Fig. 4a). In contrast, the GSH content in the Wt group reduced over time, was significantly different from the transgenic group at 8 h and at 48 h post-stimulation (*P* < 0.05). The activity expression trends of SOD and CAT were similar to each other in that both showed decreasing trends (Fig. 4b and c). The enzyme activities in the transgenic group was higher than those of the Wt group, and there were significant differences between the two groups at 1 h and 8 h post-stimulation (*P* < 0.05).

### Over-expressed toll-like receptor 2 up-regulates expression of the anti-oxidative gene heme oxygenase-1

**Fig. 5:**
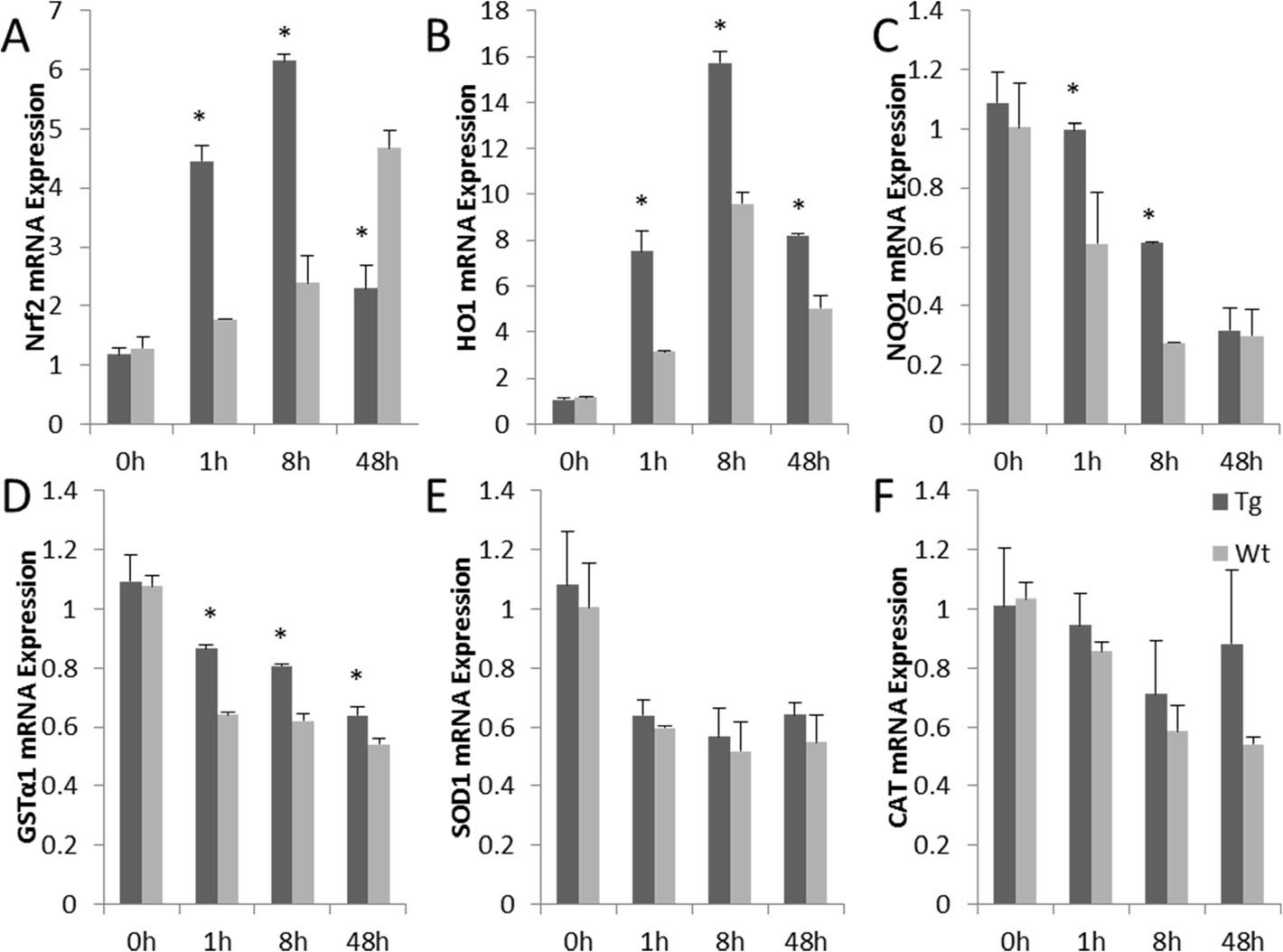
Oxidative stress gene expression patterns in goat monocytes-macrophages. **a**, **b**, **c**, **d**, **e** and **f** represent mRNA expression of *Nrf2*, *HO-1*, *NQO1*, *GSTα1*, *SOD1* and *CAT*, respectively. Tg: Transgenic goat, Wt: wild type. The results are expressed as the means ± SEM. ^*^ *P* < 0.05 in Tg versus Wt groups

Following Pam3CSK4 stimulation TLR2 over-expressed in goat monocytes-macrophages, *Nrf2* expression was significantly up-regulated at 1 and 8 h (*P* < 0.05), after which the expression level decreased, while *Nrf2* expression in the Wt group continuously increased over time (Fig. 5a). The *HO-1* expression level reached a peak at 8 h in both groups. The level of HO-1 expression in the transgenic group was significantly higher than that of the Wt at 1, 8 and 48 h post stimulation (*P* < 0.05) (Fig. 5b). Expression of the *NQO1* and *GSTα1* genes showed similar decreases over time, but were higher in Tg group than in Wt group (Fig. 5c and d). The expression levels of *SOD1* and *CAT* were not significantly different (Fig. 5e and f).

### Toll-like receptor 2 overexpression triggers infiltration of neutrophils and high-level expression of heme oxygenase-1 protein

**Fig. 6:**
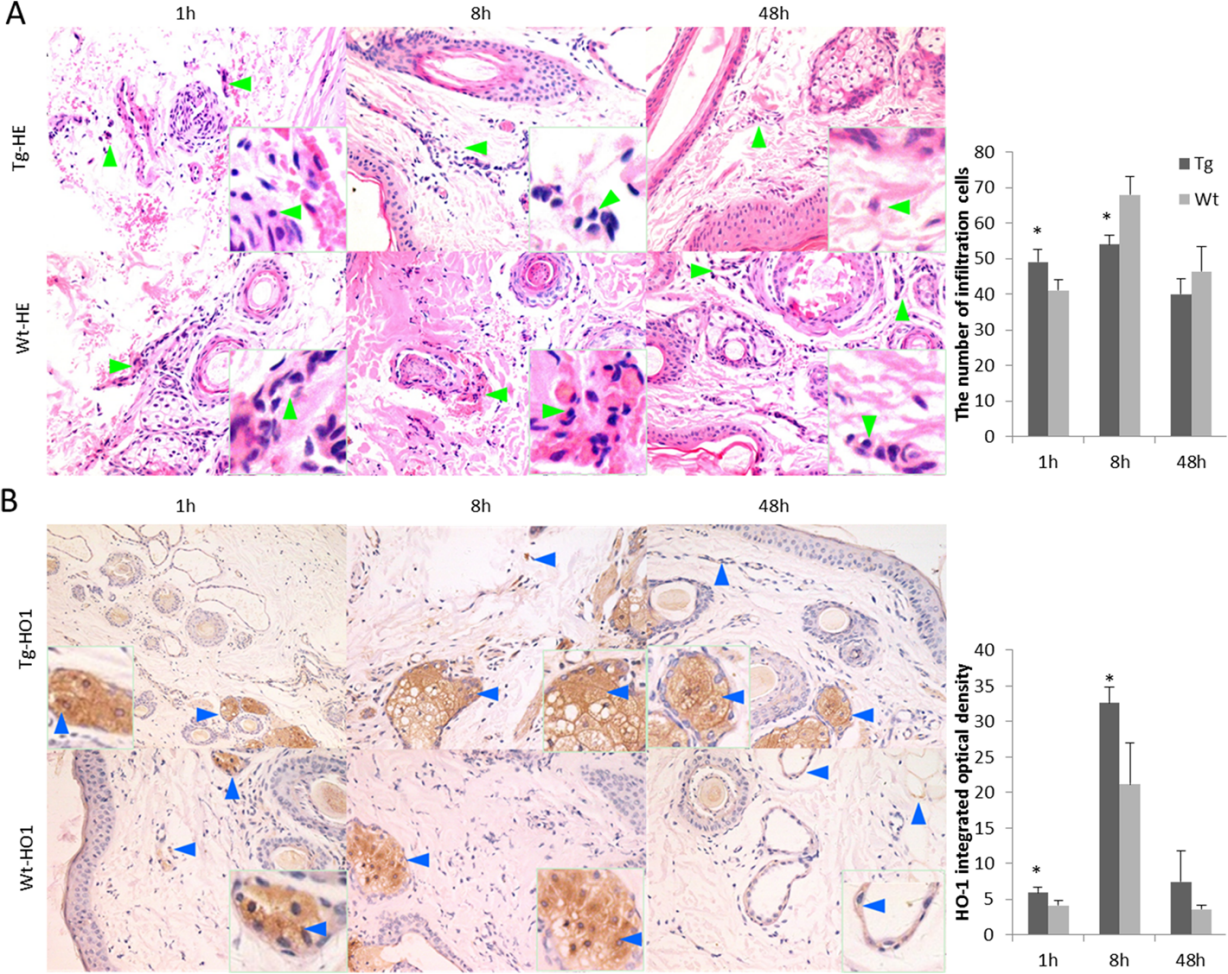
Neutrophil infiltrations in sections of dermis. **a** Pathological changes were examined microscopically (hematoxylin and eosin staining, ×400), *Green* triangle pointing to infiltration cells. **b** HO-1 expression was detected by immunohistochemical staining (×200), *Blue* triangle pointing to HO-1 positive cells. Tg: Transgenic goat, Wt: wild type. The results are expressed as the means ± SEM. ^*^ *P* < 0.05 in Tg versus Wt groups

Pam3CSK4 was injected into the ear of each transgenic goat and the resulting inflammatory infiltrate was observed by light microscopy after hematoxylin and eosin staining (Fig. 6a). In the transgenic group, a number of segmented neutrophils infiltrated the dermis at 1 h. Few infiltrating inflammatory cells were evident after 8 h. However, no significant lesions were observed after 48 h. In contrast, in the Wt group, dermal bleeding occurred in conjunction with inflammatory cell infiltration at 1 h. After 8 h, many erythrocytes were present on the skin surface and between the connective tissues and inflammatory cells, including many neutrophils that had infiltrated around the blood vessels. Few infiltrating inflammatory cells were evident after 48 h. The transgenic group displayed a strong inflammatory response at 1 h, which is consistent with the result we observed for TLR2 expression. Immunohistochemistry was used to observe HO-1 protein expression. HO-1-positive tissues showed claybank (Fig. 6b). The expression of HO-1 was maintained at a high level in transgenic group, and was significantly higher than that in the Wt group at 1 h and 8 h (*P* < 0.05).

## Discussion

Activated TLR2 triggers MyD88 protein, and I-κB kinase phosphorylation activates transcription factor NF-κB or alternatively, the MAPK pathway activates AP-1 to induce the expression of inflammatory cytokines. AP-1 is a pro-inflammation mediated transcription factor, the core components of which are c-Jun and c-Fos. Overexpression of c-Jun has been shown to induce production of inflammation mediators [20], while c-Jun/AP-1 interacts with the transcription factor, NF-κB. In cells under oxidation stress, TLR2 inhibition stopped transportation of NF-κB and AP-1 [21]. We have obtained transgenic goats with TLR2 over-expression. Excessive expression of *TLR2* mRNA was detected in monocytes-macrophages of the transgenic group. Transgenic group expressed more TLR2 protein than the Wt group in the ear tissue section [14]. In goat sera, TLR2 protein levels of the transgenic group were higher too. However without stimulation, there was no significant difference in the expression of TLR2 protein between the transgenic group and the Wt group in monocytes-macrophages. This may be caused by the negative regulation of TLRs receptors [22]. Previous study indicated, TLRs levels were kept in relative stable levels by intracellular modulation, such as alternative splice, degradation by ubiquitination and deubiquitination [23]. After Pam3CSK4-stimulation of transgenic goat monocytes-macrophages, the expression of TLR2 protein was up-regulated in the transgenic group and triggered activation of downstream transcription factors, promoted the expression of AP-1 and inhibited the activity of NF-κB. Protein levels of TLR2 in different tissues were different, and further research on the different oxidative stress response caused by it will be studied.

TNF-α and IL-1β are both able to induce the production of MCP-1. In animal experiments, it has been shown that an increase in oxidation stress can induce the expression of MCP-1 [24]. Inflammation was accompanied with an anti-inflammation reaction, and IL-4 played an important role in it. The Th1/Th2 balance is determined by two different types of cytokine secretion. The Th1 response produces IL-12 to promote the production of IFN-γ, whereas the Th2 response is distinguished by low IFN-γ levels and high IL-4 levels. TLR2 activated by different ligands generates different immune effects, and the expression of TLR2 shows time-dependence [25, 26]. It has been shown that stimulation by the TLR2 agonist Pam3CSK4 reduces the infiltration of chemokine and inflammatory cells within mouse tissues, in vivo [19]. In the present study, Pam3CSK4 could activate the monocytes-macrophages, and overexpressed TLR2 caused early expression of the pro-inflammatory cytokines TNF-α and IL-1β, and a continuous increase in expression of the anti-inflammatory factor IL-4, as well as inducing expression of Th1 type cytokines. The result of our skin inflammation experiment indicated that over-expression of the *TLR2* gene accelerated the inflammation process.

Under inflammatory conditions, free radicals are necessary for host defense. Inflammatory cells produce ROS and NO to expose the area of inflammation under oxidative stress. The transcription factors AP-1, NF-κB and Nrf2 all exhibit sensitivity to reductant-oxidant stressors. TLR2 activation of mouse macrophages produces NO resulting in damage to bacteria within cells [27], while NADPH oxidase regulates the production of ROS, over-expresses pro-inflammatory cytokines, and positively facilitates oxidative stress. Within cells, there is both elevation of ROS and activation of NF-κB. The production of NO is regulated by iNOS, and the activation of Nrf2 can reduce induction of iNOS expression by IL-1β and inhibit the activity of NF-κB [28]. The results of the present study showed that Pam3CSK4 stimulated goat monocytes-macrophages, and over-expressed TLR2 up-regulated the activity of NADPH oxidase. Furthermore, the low activity of iNOS in the TLR2 over-expressed group showed that NO was synthesized in small amounts, and that the oxidative stress injury to cells was weak. Over-expressed *TLR2* caused the over-expression of Nrf2 and inhibited iNOS activity. It has been shown that an Nrf2 agonist inhibited TNF-α and IL-1β and induced COX-2 expression by blocking the activation of NF-κB [29]. High expression of COX-2 inhibited PI3K activity and the anti-oxidation reaction mediated by Nrf2 [30]. In addition, activation of Nrf2 can regulate c-Jun signal activity directly, and COX-2 expression negatively. Our results of showed that over-expressed TLR2 induced the up-regulation of Nrf2, increased the expression of the *c-Jun* gene, and inhibited the expression of COX-2 in monocytes-macrophages under Pam3CSK4 stimulation.

Too many free radicles can disturb the balance between the oxidation and anti-oxidation systems of a host. GSH, SOD and CAT are important anti-oxidative substances in a host organism. Over-expression of anti-oxidants can block NF-κB activation. Also, activated NF-κB can induce secretion of pro-inflammatory factors, reduce SOD activity and increase the MDA content of a cell [31]. Our results indicate that the consumption of anti-oxidation stress enzymes in TLR2 over-expressed cells was lower than that of the Wt cells, thereby effectively maintaining the oxidative stress balance of the cells. The result of this study also shows that TLR2 over-expression in monocytes-macrophages stimulated by Pam3CSK4 improved GSH activity and that the GSH consumed could be synthesized rapidly.

We also found that oxidative stress activated NF-κB and AP-1 leading to the production of pro-inflammatory cytokines, while excessive oxidative stress activated the Nrf2 signal pathway [32]. Nrf2-induced HO-1 expression was found to inhibit the activation of NF-κB and the secretion of MCP-1 in endothelial cells stimulated by TNF-α [33]. Additionally, expression of iNOS in Nrf2 knockout mice was significantly higher than that seen in Wt mice [34]. Here, at 8 h post-stimulation with Pam3CSK4, TLR2 over-expression in transgenic goat monocytes-macrophages was found to up-regulate expression of the Nrf2 gene. Expression of the pro-inflammatory factors TNF-α decreased. The activity of iNOS was lower than that of the control. Importantly, the transgenic group experienced a weaker inflammatory reaction than that of the control group by the ear skin regional inflammation test. It indicates that TLR2 up-regulated expression of the *Nrf2* gene, thereby reducing the level of oxidative injury in the host.

AP-1 family members include Jun and Fos. Jun subclasses include c-jun and JunB. Fos subclasses include c-fos and FosB. Different types of AP-1 transcription factor dimer combinations have different functions in gene expression regulation. AP-1 positively regulates the expression of most genes, but increased levels of its c-Fos and c-Jun subunits negatively regulate expression of some genes [35]. The NQO1 gene ARE contains an AP-1 binding site. NQO1 induced by ARE is positively regulated by Nrf2 and negatively regulated by c-Fos [36]. NQO1 and GST are highly expressed in c-Fos knockout mice [37], and our results are consistent with these findings. Over-expressed TLR2 up-regulated the expression of *AP-1* and its *c-Fos* and *c-Jun* gene subunits; indeed, their expression trends were similar. Expression of the anti-oxidation genes *NQO1*, *GSTα1*, *SOD1* and *CAT* were inhibited.

HO-1 is a rate-limiting enzyme in heme catalysis and the protection system comprising HO-1–bilirubin–carbon monoxide (CO) widely participate in anti-inflammatory reactions and oxidative stress injuries. Similar to NO, CO was also found to reduce the production of inflammatory factors [38]. Researchers have discovered that activation of the *HO-1* gene inhibited the activity of the AP-1 protein [39]. However, many studies have shown that AP-1 is involved in the induction of HO-1 expression [40, 41]. In Nrf2 knockout cells, nitrite improved the activity of AP-1 through the JNK/c-Jun pathway and up-regulated HO-1 expression. Lipoteichoic acid induced expression of the *HO-1* gene through the TLR2/MyD88/c-Src/NADPH oxidase pathway in tracheal smooth muscle cells [42]. The results of our study showed that, in Pam3CSK4 stimulated goat monocytes-macrophages, the expression of AP-1 and PI3K were up-regulated in the TLR2 over-expression group; this facilitated expression of the *Nrf2* gene and induced an increase in *HO-1* gene expression.

## Conclusions

Our study showed that, stimulated by the synthetic bacterial lipoprotein Pam3CSK4, TLR2 over-expressed in goat monocytes-macrophages triggered the expression of the anti-oxidation gene *Nrf2*, up-regulated expression of the *HO-1* gene, inhibited an excessive immuno-response, and enhanced the anti-oxidative stress injury in the host through activating the AP-1 and PI3K signal pathways. TLR2 over-expressed in goats might reduce the inflammatory and oxidative stress damage.
