# Erratum to: Over-expression of Toll-like receptor 2 up-regulates heme oxygenase-1 expression and decreases oxidative injury in dairy goats

**DOI:** 10.1186/s40104-017-0149-5

**Published:** 2017-02-15

**Authors:** Shoulong Deng, Kun Yu, Wuqi Jiang, Yan Li, Sutian Wang, Zhuo Deng, Yuchang Yao, Baolu Zhang, Guoshi Liu, Yixun Liu, Zhengxing Lian

**Affiliations:** 1 State Key Laboratory of Stem Cell and Reproductive Biology, Institute of Zoology, Chinese Academy of Sciences, Beijing, 100101 China; 2 Laboratory of Animal Genetics and Breeding, College of Animal Science and Technology, China Agricultural University, Beijing, 100193 People’s Republic of China; 3 National key Lab of Agrobiotechnology, College of Biological Sciences, China Agricultural University, Beijing, 100193 People’s Republic of China; 4 Department of Animal Science, Oklahoma State University, Stillwater, OK 74078 USA; 5 College of Animal Science and Technology, Northeast Agricultural University, Harbin, 150030 People’s Republic of China; 6 State Oceanic Administration, Beijing, 100860 People’s Republic of China

## Erratum

Following publication of this article [1], it has come to our attention that the name of the author Sutian Wang’s name was captured incorrectly as “Shuotian Wang” and instead should be Sutian Wang.

## References

[1] Deng S, Yu K, Jiang W, Li Y, Wang S, Deng Z, Yao Y, Zhang B, Liu G, Liu Y, Lian Z (2017). Over-expression of Toll-like receptor 2 up-regulates heme oxygenase-1 expression and decreases oxidative injury in dairy goats. J Anim Sci Biotechnol.

